# Aligning Post-Column ESI-MS, MALDI-MS, and Coagulation Bioassay Data of *Naja* spp., *Ophiophagus hannah*, and *Pseudonaja textillis* Venoms Chromatographically to Assess MALDI-MS and ESI-MS Complementarity with Correlation of Bioactive Toxins to Mass Spectrometric Data

**DOI:** 10.3390/toxins16090379

**Published:** 2024-08-29

**Authors:** Haifeng Xu, Susan El-Asal, Hafsa Zakri, Rama Mutlaq, Natascha T. B. Krikke, Nicholas R. Casewell, Julien Slagboom, Jeroen Kool

**Affiliations:** 1 Amsterdam Institute of Molecular and Life Sciences, Division of BioAnalytical Chemistry, Department of Chemistry and Pharmaceutical Sciences, Faculty of Science, Vrije Universiteit Amsterdam, De Boelelaan 1085, 1081HV Amsterdam, The Netherlands; 2 Centre for Analytical Sciences Amsterdam (CASA), 1012 WX Amsterdam, The Netherlands; 3 Centre for Snakebite Research and Interventions, Department of Tropical Disease Biology, Liverpool School of Tropical Medicine, Liverpool L3 5QA, UK

**Keywords:** plotted matrix-assisted laser desorption ionization (MALDI-MS) chromatograms, electrospray ionization (ESI) MS-extracted ion currents (EICs), plasma coagulation bioassay chromatograms, nanofractionation analytics, chromatographic alignment

## Abstract

Snakebite is a serious health issue in tropical and subtropical areas of the world and results in various pathologies, such as hemotoxicity, neurotoxicity, and local swelling, blistering, and tissue necrosis around the bite site. These pathologies may ultimately lead to permanent morbidity and may even be fatal. Understanding the chemical and biological properties of individual snake venom toxins is of great importance when developing a newer generation of safer and more effective snakebite treatments. Two main approaches to ionizing toxins prior to mass spectrometry (MS) analysis are electrospray ionization (ESI) and matrix-assisted laser desorption ionization (MALDI). In the present study, we investigated the use of both ESI-MS and MALDI-MS as complementary techniques for toxin characterization in venom research. We applied nanofractionation analytics to separate crude elapid venoms using reversed-phase liquid chromatography (RPLC) and high-resolution fractionation of the eluting toxins into 384-well plates, followed by online LC-ESI-MS measurements. To acquire clear comparisons between the two ionization approaches, offline MALDI-MS measurements were performed on the nanofractionated toxins. For comparison to the LC-ESI-MS data, we created so-called MALDI-MS chromatograms of each toxin. We also applied plasma coagulation assaying on 384-well plates with nanofractionated toxins to demonstrate parallel biochemical profiling within the workflow. The plotting of post-column acquired MALDI-MS data as so-called plotted MALDI-MS chromatograms to directly align the MALDI-MS data with ESI-MS extracted ion chromatograms allows the efficient correlation of intact mass toxin results from the two MS-based soft ionization approaches with coagulation bioassay chromatograms. This facilitates the efficient correlation of chromatographic bioassay peaks with the MS data. The correlated toxin masses from ESI-MS and/or MALDI-MS were all around 6–8 or 13–14 kDa, with one mass around 20 kDa. Between 24 and 67% of the toxins were observed with good intensity from both ionization methods, depending on the venom analyzed. All *Naja* venoms analyzed presented anticoagulation activity, whereas pro-coagulation was only observed for the *Pseudonaja textillis* venom. The data of MALDI-MS can provide complementary identification and characterization power for toxin research on elapid venoms next to ESI-MS.

## 1. Introduction

Snakebite exerts a devastating toll, causing approximately 15,400–57,600 deaths in Asia and 3500–32,100 deaths in sub-Saharan Africa alone each year [1]. Additionally, it leads to more than 400,000 individuals suffering amputations or enduring other permanent disabilities annually, with a disproportionate impact in tropical rural areas where antivenom availability and affordability are limited [2]. The pathophysiological consequences of snakebites are broadly categorized into neurotoxicity, hemotoxicity, and cytotoxicity, resulting in neuromuscular paralysis, hemorrhage or coagulopathy, and blistering or tissue necrosis around the bite site, respectively [3].

Snake venoms are complex mixtures comprising proteins, peptides, lipids, carbohydrates, amines, and various small molecules. Major protein families include secreted phospholipases A_2_ (PLA_2_s), snake venom metalloproteinases (SVMPs), snake venom serine proteases (SVSPs), and three-finger toxins (3FTXs), with the minor families encompassing cysteine-rich secretory proteins (CRISPs), L-amino acid oxidases (LAAOs), Kunitz peptides, C-type lectins, disintegrins, and natriuretic peptides [4,5]. The composition and relative proportion of venom toxins exhibit substantial variation due to inter- and intraspecific [6,7], geographical [8], age-related [9], sex-related [10], dietary [11], and seasonal [12] differences. These variations impose significant challenges in the context of snakebite treatment, including restricting the efficacy of current therapies to certain snake species [13,14,15]. Therefore, a deeper understanding of venom toxins, their variability, and their envenoming mechanisms is essential for the development of newer, safer, and more affordable snakebite treatments. Furthermore, this knowledge could help in better understanding snake evolution [16] and potentially help in the effort to discover novel biopharmaceuticals derived from venom toxins [17,18].

To achieve comprehensive toxin identification and quantification, venomics studies, which encompass venom proteomics and may include genomics, transcriptomics, and bioinformatics analyses, can be performed to provide extensive insights into venom composition [19,20,21,22]. Nanofractionation analytics as an analytical methodology for snake venom profiling has emerged over the last few years [23,24,25,26]. It encompasses LC separation with UV detection, followed by high-resolution fraction collection on high-density (e.g., 384-well) well plates and parallel online MS detection enabled via a post-column split. The methodology was developed for post-column measurements of certain bioactivities of the fractionated toxins by applying a downstream bioassay of choice post-fractionation. The parallel MS data provide chemical information on the bioactive toxins in terms of their accurate masses. ESI is commonly employed for this approach of online MS analysis, enabling the direct ionization of eluting toxins after LC separation, which allows for subsequent mass analysis to determine analyte molecular weights and other structural characteristics [27,28,29,30]. This, in turn, when using normal ESI-Q-TOF (Quadrupole-Time of Flight)-MS coupled to normal-bore LC separations, which is often used for this, results in sensitivity that typically decreases when a toxin mass increases [31].

To achieve more comprehensive and efficient identification of not only the smaller toxins in venoms but also the larger toxins, the use of MALDI-MS presents an interesting option, which has demonstrated applicability in identifying high-molecular-mass toxins and assigning PTMs (post-translated modifications) [32,33,34], for the characterization of purified snake toxins [35] and for venomics profiling [29]. In short, the MALDI-matrix crystallized analyte is shot with a nanosecond laser beam of a UV laser with wavelengths of 266 or 337 nm, resulting in sublimation and ionization, thereby producing the protonated or deprotonated analyte. Depending on the analytes to be analyzed, different matrixes and solutions or sample preparations for analyte crystallization can be selected [36,37]. The large differences in the principle of ionization for ESI and MALDI also result in different data obtained when analyzing proteins such as venom toxins. Detection by ESI-MS when using a Q-TOF mass spectrometer gives accurate mass ion information of all charge states of each eluting peak directly online and analyzed after LC separation. In snake venom research, MALDI-MS ions are observed as singly charged ions and, sometimes, as doubly charged ions as well.

Rather than relying solely on a single analytical method for toxin identification, researchers are increasingly inclined to employ multiple analytics in parallel to acquire more detailed and comprehensive information, including N-terminal sequencing and cysteine profiling [38], identification of isoforms with corresponding unique peptides [39], and PTM determinations [40]. Studies have also used both ESI-MS and MALDI-MS to investigate proteins, such as studies dealing with DNA-binding proteins [41], the rat urinary proteome [42], patient saliva [43], honeybee venom [44], and spider venom [45]. In our study, we focus on the venoms of elapid snakes and compare the results of both approaches by aligning post-column ESI-MS and MALDI-MS data chromatographically. Combining data on separated venom toxins from both ESI-MS and MALDI-MS data presents a more comprehensive MS analysis strategy that will likely yield mass spectral data on more toxins than when using only one of the two MS methods. The separation of venom toxins from crude venoms followed by ESI-MS can be performed using standard LC-ESI-Q-TOF-MS equipment. As discussed, for MALDI-MS, which is an offline MS technique, after LC, the separated toxins first need to be fractionated and crystallized with the MALDI matrix prior to offline MALDI-MS analysis and post-column coagulation bioassay [46,47]. The present work investigates the combined use of the two ionization approaches, ESI and MALDI, for the analysis of elapid venom toxins after LC separation.

## 2. Results and Discussion

This study presents and discusses the processed results from LC-UV, LC-ESI-MS, LC-MALDI-MS, and post-column coagulation bioactivity assaying of several medically relevant elapid venoms by nanofractionation analytics. The superimposed chromatographic datasets allow clear comparisons and alignment of bioassay peaks with mass spectrometry data. Toxins found with both MS techniques are named “matched” toxins in this study, and the ones detected only in MALDI-MS data are named “unmatched” toxins. The matched and unmatched masses found for toxins from elapid venoms were analyzed, and the coagulation activity assessment of the nanofractionated toxins incorporated demonstrated parallel biochemical profiling within the experimental workflow.

**Figure 1:**
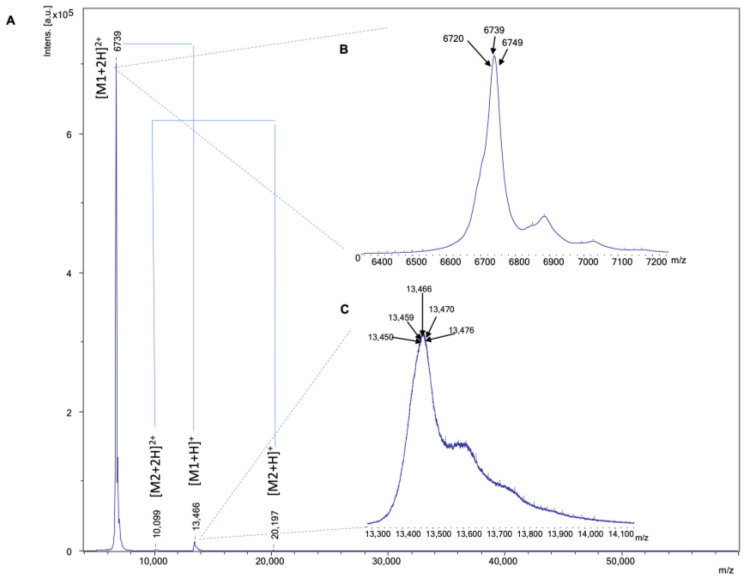
(**A**) A typical MALDI-MS (MS1) spectrum of a venom toxin. The *m*/*z* range of 5000–50,000 was plotted. The toxin MS spectrum measured was from the fraction in well B17 with an elution retention time of 26.4 min during the nanofractionation of the *N. kaouthia* venom. The toxin’s singly charged (M+H)^+^ and doubly charged (M+2H)^2+^ ions (which were both used for toxin mass deconvolution) are shown. (**B**,**C**) Zoomed-in toxin ion peaks of the toxin. The *m*/*z* of 6739 and 13,466 are the doubly and singly charged ions of the toxin, respectively. The calculated toxin’s accurate mass was 13,466 Da.

To obtain the masses of the fractionated toxins from the MALDI-MS data, the MALDI-MS spectra obtained from each fraction shown to contain signals (focus was placed on the fractions that were fractionated during the elution times where the LC-UV data showed eluting toxins) were manually investigated. Then, the *S*/*N* of the toxin ion peaks and ion masses were retrieved by the software. Figure 1A shows a typical MALDI-MS spectrum obtained from a nanofractionated toxin in the well, with a retention time of fractionation of 26.4 min, resulting from analysis of *Naja kaouthia* venom. The toxin ions presented themselves as singly (M+H)^+^ and doubly charged (M+2H)^2+^ ions (which were used for toxin mass deconvolution). Figure 1B,C show zoomed-in toxin ion peaks of two observed ions with *m*/*z* of 6739 and 13,466 of the doubly and singly charged ions, respectively. If only one ion was found in the MALDI-MS data and if the ion was also observed in ESI-MS, the parallel acquired ESI-MS data were used to assess the charge state of the *m*/*z*-value found in the MALDI-MS data. The figure shows that the broad ion peaks in MALDI-MS gave masses that were rounded to the integer mass number. As anticipated, with ESI-MS, much higher resolutions were achieved. A general mass error in MALDI-MS of <10 Da was used in this study. This range of the allowed mass error offset was also used/acknowledged by Schiller et al. [48], who used a maximum cutoff of around 16 Da when measuring lipids (1-Palmitenyl-2-hydroxy-PC, (M+H)^+^) with MALDI-TOF-MS. Another example can be found in the analysis of the *Walterinnesia aegyptia* snake venom by MALDI-TOF-MS by Aziz et al., who used a maximum mass error of 8 [49]. A mass error of 11 Da was allowed in the mass fingerprints from MALDI-TOF-MS analyses of crude venom from *Atheris squamigera* [50]. Wermelinger et al. set the mass difference to fall within the uncertainty range at ±0.05%, resulting in assigning, for example, the ions *m*/*z* 7741 and 7732 found in both *B. insularis* and *B. jararaca* venoms, respectively, as having the same molecular mass [51]. In addition, Newton et al. used a maximum *m*/*z* difference of 8 (<0.1%) for the mass fingerprinting of post-column collected fractions from the venom of the Indian red scorpion (*Mesobuthus tamulus*) [52]. For the MALDI-MS analysis of *O. cayaporum*, the authors used a Da error of less than 8 when matching results between ESI and MALDI [53]. In another study that looked at enteric bacteria, a maximum error of below 7 Da was used to compare the results from ESI and MALDI [54]. We selected a mass error below 10 Da to compare the results from ESI-MS and MALDI-MS.

The integrated analytical results obtained in this study are presented and discussed in the next sections, ordered by the snake venom analyzed.

### 2.1. Naja kaouthia

**Figure 2:**
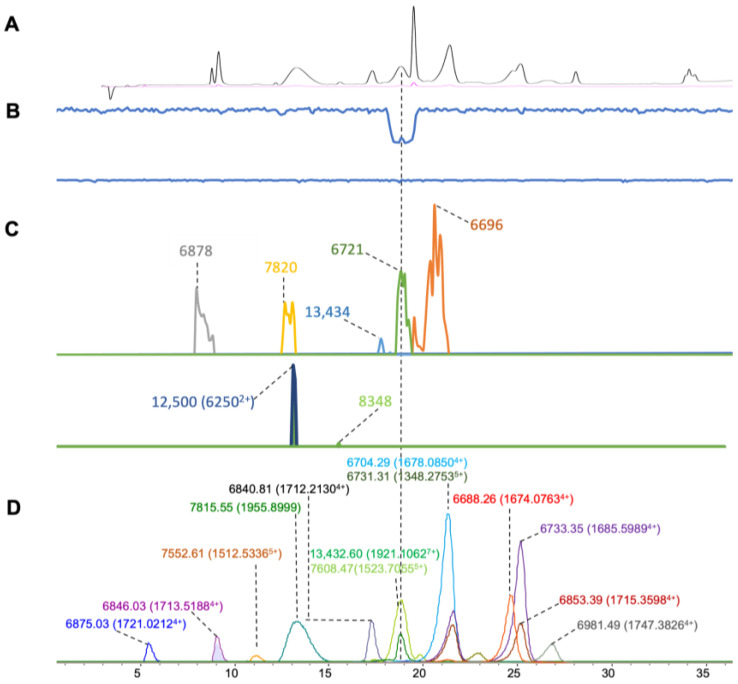
Integrated *N. kaouthia* venom results: Superimposed results of LC-UV data, LC-(ESI)-MS data, nanofractionation MALDI-MS data plotted chromatographically, and nanofractionation coagulation bioassay data plotted chromatographically. (**A**) LC-UV trace of separated snake venom at 220 nm (black) and 254 nm (purple). (**B**) Coagulation bioactivity chromatograms representing anticoagulation (upper trace) and pro-coagulation activity (lower trace). (**C**) Chromatographically plotted MALDI-MS data of the identified toxins in the wells with nanofractionated toxins. For each toxin identified in different wells, the measured intensity from the MALDI data was plotted on the y-axis and the retention time of fractionation was plotted on the x-axis. As all the toxins were eluted over a series of subsequent wells, so-named MALDI-MS chromatograms of each toxin were obtained. Two sub-graphs are plotted in (C) with y-axis ranges of 0–4000 (high) and 0–80 (low). (**D**) Extracted Ion Currents (EICs) from the LC-ESI-MS data.

For the *N. kaouthia* venom, MS signals representing a total of thirteen masses were recognized with high intensity from the ESI-MS measurement for our comparative study. For each charge state envelope representing a toxin’s accurate mass, the highest abundant charge state was selected, and then, its EIC was plotted (Figure 2D). Most toxin masses included for comparison were around 6–7 kDa, with only one toxin with a mass around 13 kDa. The toxin masses around 6–7 kDa most likely corresponded to 3FTXs and the toxin with the mass around 13 kDa presumably is a PLA_2_ [55]. For the so-named MALDI-MS chromatograms (Figure 2C), we plotted seven clearly observed masses retrieved from the fractionated toxins for our comparative study (the accurate masses calculated from high-resolution ion peaks were generated by the MS software(v 6.0). Among those, four masses were matched with the masses of the ESI-MS results, with mass errors lower than around ±10 Da (Table 1). In addition, nine accurate masses with accompanying distinct retention times were found with the restrictions set in terms of the signal intensity for the ESI-MS measurements. When looking at the bioassay results, a notable anticoagulation activity (Figure 2B) was observed at a retention time of 13.5 min, corresponding to a toxin mass of 13,432.60 and 13,434 Da retrieved from ESI-MS and MALDI-MS, respectively. A PLA_2_ toxin from *N. kaouthia* (Uniprot ID, P00596) was found to have a similar exact mass (13,432.70 Da) with an anticoagulant activity [56] and inhibitory activity on nicotinic acetylcholine receptors [57]. In addition to this PLA_2_ toxin, two 3FTX-like toxins (6840.81 and 7608.47 Da) were deconvoluted from the ESI-MS data that co-eluted, which were likely the same as those found in another study where toxin masses of 6839.5 and 7615.3 Da were assigned for crude *Naja kaouthia* venom analysis by LC-ESI-MS [58].

### 2.2. Naja naja

**Figure 3:**
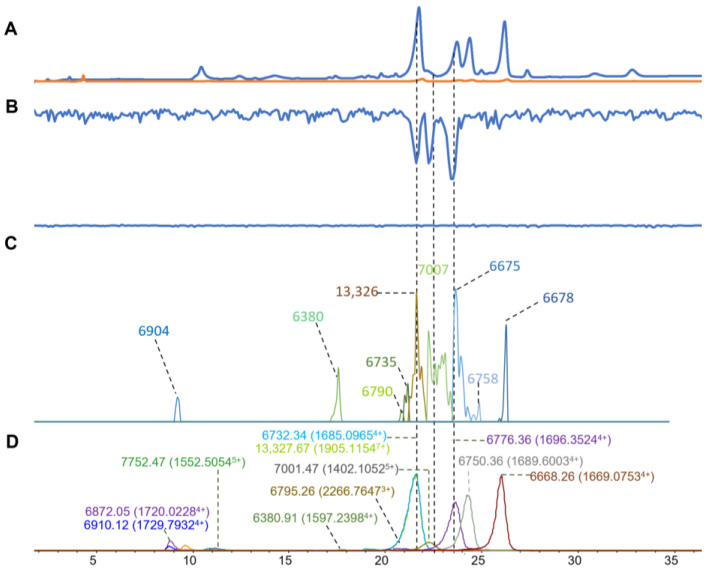
Integrated *N. naja* venom results: Superimposed results of LC-UV data, LC-(ESI)-MS data, nanofractionation MALDI-MS data plotted chromatographically, and nanofractionation coagulation bioassay data plotted chromatographically. (**A**) LC-UV trace of separated snake venom at 220 nm (blue) and 254 nm (orange). (**B**) Coagulation bioactivity chromatograms representing anticoagulation (upper trace) and pro-coagulation activity (lower trace). (**C**) Chromatographically plotted MALDI-MS data of the identified toxins in the wells with nanofractionated toxins. For each toxin identified in different wells, the measured intensity from the MALDI data was plotted on the y-axis and the retention time of fractionation was plotted on the x-axis. As all the toxins were eluted over a series of subsequent wells, so-named MALDI-MS chromatograms of each toxin were obtained. Sub-graph plotted in (**C**) with y-axis range of 0–6000 (high). (**D**) Extracted Ion Currents (EICs) from the LC-ESI-MS data.

For the *N. naja* venom, fourteen high-intensity masses were detected from the ESI-MS measurement. Similar to the *N. kaouthia* venom, most masses were around 6–7 kDa, with one mass around 13 kDa (Table 1). From the MALDI-MS results (Figure 3C), we found fifteen masses with good MS ion intensity. Nine of those masses could be matched with a toxin mass found in the ESI-MS data (Table 1). For the results of the coagulation bioassay, we found three negative peaks represented as anticoagulation in the retention times around 21.5, 22.5, and 23.5 min (Figure 3B). The corresponding retention time matching masses from the ESI-MS data were 13,327.67 and 6732.34, 7001.47, and 6676.36 Da. These masses were also found from the MALDI-MS data for which the following masses were matched: 13,326 and 6735, 7007, and 6675 Da. It was suggested that these toxins were 3FTXs and PLA_2_s based on their mass ranges and previous proteomic research on *N. naja* venom [59]. The ESI-MS toxin mass of 13,327.67 Da (in MALDI-MS 13,326 Da) was tentatively matched to a PLA_2_ toxin (Uniport ID, PA2A2_NAJNA) with the exact mass of 13,322.64 Da. In other anticoagulation studies focusing on *N. naja* venom, PLA_2_s were recognized as predominant toxins that contributed to anticoagulation bioactivity [60,61].

### 2.3. Naja haje

**Figure 4:**
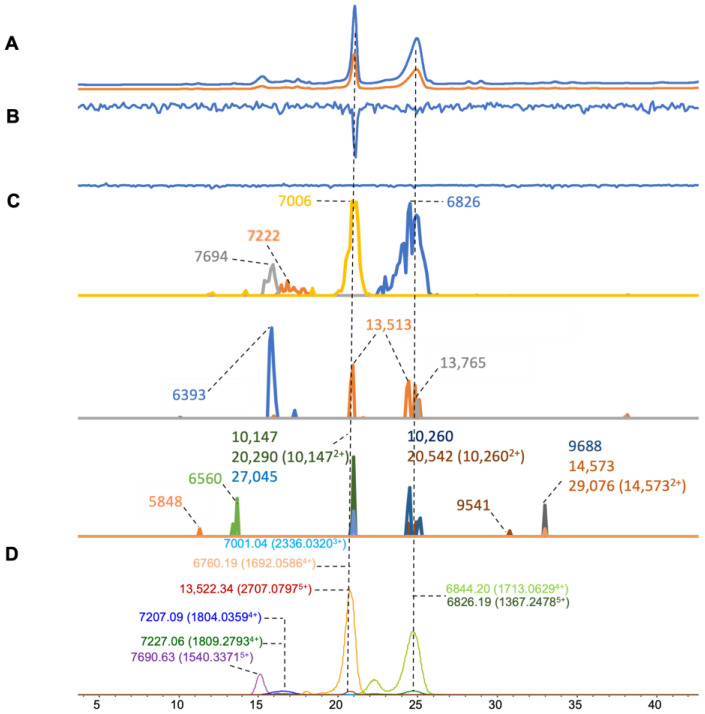
Integrated *N. haje* venom results: Superimposed results of LC-UV data, LC-(ESI)-MS data, nanofractionation MALDI-MS data plotted chromatographically, and nanofractionation coagulation bioassay data plotted chromatographically. (**A**) LC-UV trace of separated snake venom at 220 nm (blue) and 254 nm (orange). (**B**) Coagulation bioactivity chromatograms representing anticoagulation (upper trace) and pro-coagulation activity (lower trace). (**C**) Chromatographically plotted MALDI-MS data of the identified toxins in the wells with nanofractionated toxins. For each toxin identified in different wells, the measured intensity from the MALDI data was plotted on the y-axis and the retention time of fractionation was plotted on the x-axis. As all the toxins were eluted over a series of subsequent wells, so-named MALDI-MS chromatograms of each toxin were obtained. Three sub-graphs are plotted in (**C**) with y-axis ranges of 0–10,000 (high), 0–600 (medium) and 0–100 (low). (**D**) Extracted Ion Currents (EICs) from the LC-ESI-MS data.

For the venom of *N. haje*, nine good-intensity toxin masses were found in the ESI-MS data, with a mass range of 6–14 kDa (Figure 4D), whereas sixteen toxin masses were retrieved from the MALDI-MS data (Figure 3C). Among the deconvoluted masses from the MALDI-MS data, three doubly charged ions were found and deconvoluted (*m*/*z* of 10,147, 10,260, and 14,573), with the resulting accurate masses of 20,290, 20,542, and 29,076 Da respectively. Five masses found in MALDI-MS could be matched with the data from ESI-MS (Table 1). From the results of the coagulation bioassay, notable anticoagulation activity was observed in the coagulation chromatogram between 20 to 21 min corresponding to masses in both ESI-MS (7001.04 and 13,522.33 Da) and MALDI-MS (7006 and 13,513 Da). The toxin with a mass of around 13.5 kDa was likely an anticoagulant PLA_2_ (Figure 4B). In 2010, Osipov et al. isolated a PLA_2_ toxin named TI-Nh (accurate mass of 14,340 Da) from *N. haje* cobra venom that could inhibit fibrinogenolytic activity and induce platelet aggregation [62]. Venom was separated and toxins were purified by gel filtration, cation-exchange HPLC, and reversed-phase HPLC following identification by the MALDI-MS of intact toxins and by bottom-up proteomics (i.e., after tryptic digestion). However, the mass of TI-Nh was not found in our analyzed venom.

### 2.4. Naja atra

**Figure 5:**
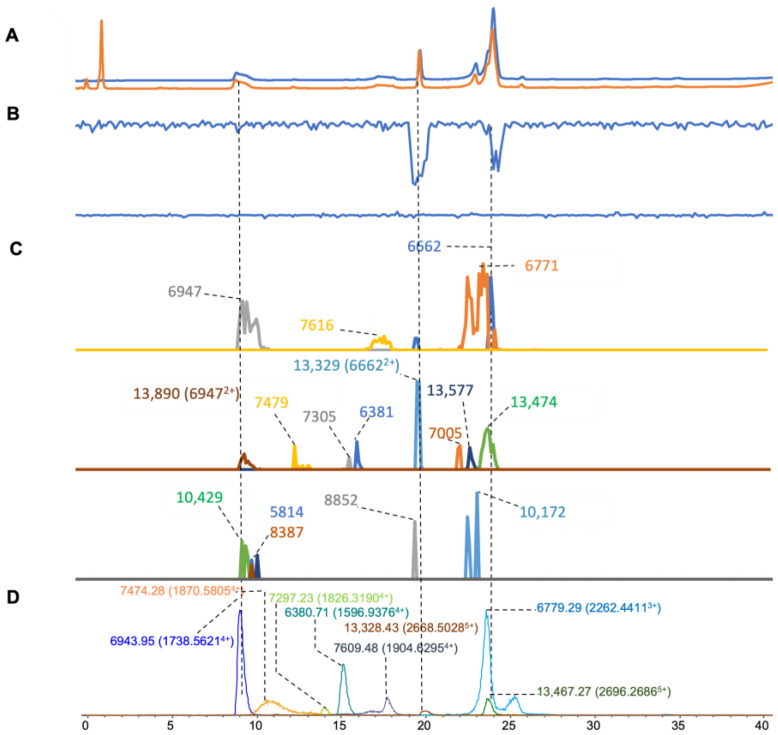
Integrated *N. atra* venom results: Superimposed results of LC-UV data, LC-(ESI)-MS data, nanofractionation MALDI-MS data plotted chromatographically, and nanofractionation coagulation bioassay data plotted chromatographically. (**A**) LC-UV trace of separated snake venom at 220 nm (blue) and 254 nm (orange). (**B**) Coagulation bioactivity chromatograms representing anticoagulation (upper trace) and pro-coagulation activity (lower trace). (**C**) Chromatographically plotted MALDI-MS data of the identified toxins in the wells with nanofractionated toxins. For each toxin identified in different wells, the measured intensity from the MALDI data was plotted on the y-axis and the retention time of fractionation was plotted on the x-axis. As all the toxins were eluted over a series of subsequent wells, so-named MALDI-MS chromatograms of each toxin were obtained. Three sub-graphs are plotted in (**C**) with y-axis ranges of 0–8000 (high), 0–1000 (medium) and 0–20 (low). (**D**) Extracted Ion Currents (EICs) from the LC-ESI-MS data.

In the venom of *N. atra,* eight mass ions were found in the ESI-MS data (Figure 5D) (mass range of 6–13 kDa). Sixteen apparent masses were found in the data of the MALDI-MS measurements (Figure 5C). Among those, eight masses were matched for both MS methods and were in the mass range of 6.5 to 13.8 kDa (Table 1). In the results from the coagulation bioassay, anticoagulation activity with two notable negative peaks was observed in the anticoagulation chromatogram. The first anticoagulation peak was at 20 min, corresponding to the mass of 13,328.42 and 13,329 in ESI-MS and MALDI-MS, respectively (Figure 5B). The second anticoagulation peak was at 24 min and corresponded to the masses of 6779.29 and 13,467.26 or 6771 and 13,474 in ESI-MS and MALDI-MS, respectively. Based on our data, it is likely that a PLA_2_ caused the observed anticoagulant activity.

### 2.5. Naja pallida

**Figure 6:**
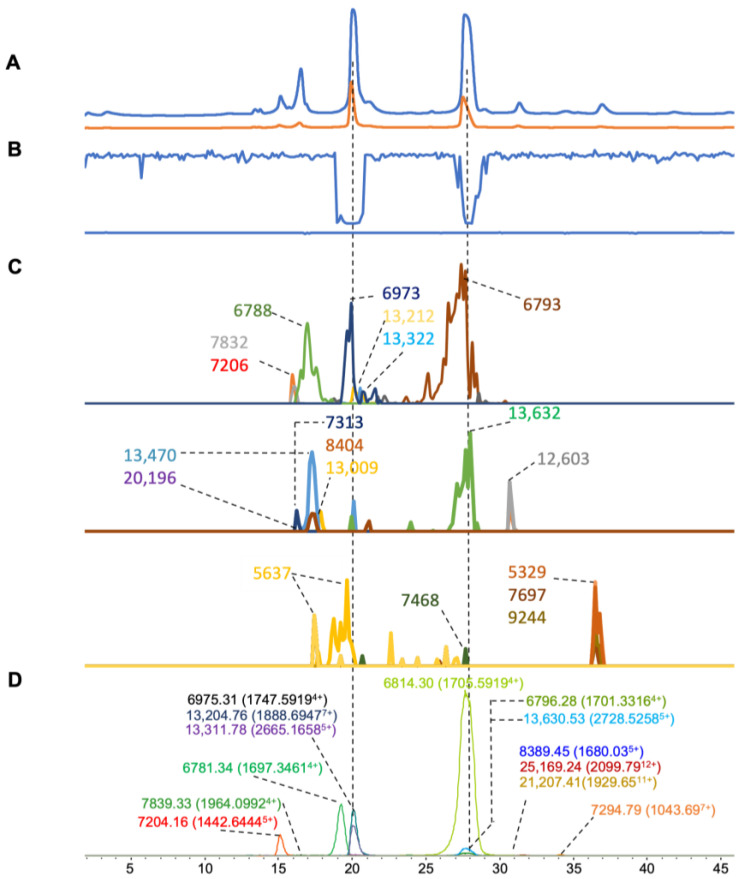
Integrated *N. pallida* venom results: Superimposed results of LC-UV data, LC-(ESI)-MS data, nanofractionation MALDI-MS data plotted chromatographically, and nanofractionation coagulation bioassay data plotted chromatographically. (**A**) LC-UV trace of separated snake venom at 220 nm (blue) and 254 nm (orange). (**B**) Coagulation bioactivity chromatograms representing anticoagulation (upper trace) and pro-coagulation activity (lower trace). (**C**) Chromatographically plotted MALDI-MS data of the identified toxins in the wells with nanofractionated toxins. For each toxin identified in different wells, the measured intensity from the MALDI data was plotted on the y-axis and the retention time of fractionation was plotted on the ***x***-axis. As all the toxins were eluted over a series of subsequent wells, so-named MALDI-MS chromatograms of each toxin were obtained. Three sub-graphs are plotted in (C) with y-axis ranges of 0–10,000 (high), 0–600 (medium) and 0–60 (low). (**D**) Extracted Ion Currents (EICs) from the LC-ESI-MS data.

For the venom of *N. pallida* (Figure 6), fourteen clear mass ions with highly abundant signals were found in the ESI-MS data, with a mass range of 6–25 kDa (Figure 6D), whereas nineteen clear mass ions were found in the MALDI-MS data (Figure 6C). There were 11 matched masses from both MS methods ranging from 6 to 21 kDa (Table 1). Two significant anticoagulation peaks were observed at retention times of around 20 and 28 min (Figure 6B). In the retention time range of 19 to 21 min, one toxin was found with a mass of 6975.30 and 6973 Da in ESI-MS and MALDI-MS, respectively, and a second mass was deconvoluted with a mass of 13,204.76 and 13,212 Da in ESI-MS and MALDI-MS, respectively. Another mass was deconvoluted as 13,311.78 and 13,322 Da in ESI and MALDI, respectively. Likely, toxins around 13 kDa, i.e., in the PLA_2_ mass range, caused the anticoagulation observed. Around the retention time of 28 min, three ions (6814.30, 6796.28, and 13,630.53 Da) were found in ESI-MS. Regarding MALDI-MS around the same retention time, two toxins were deconvoluted as 6793 and 13,632 Da. It is likely that the resolution of the MALDI-MS measurements prevented the discrimination of the two very similar masses of the toxins measured by ESI-MS with accurate masses of 6814.30 and 6796.28 Da. We found one PLA_2_ (Uniport ID: PA2B_NAJPA) with the exact mass of 13,317.14 Da in UniProt with significant anticoagulant activity [47], which matched with our accurate mass deconvoluted data from both ESI-MS (13,311.78 Da) and MALDI-MS (13,322 Da).

### 2.6. Ophiophagus hannah

**Figure 7:**
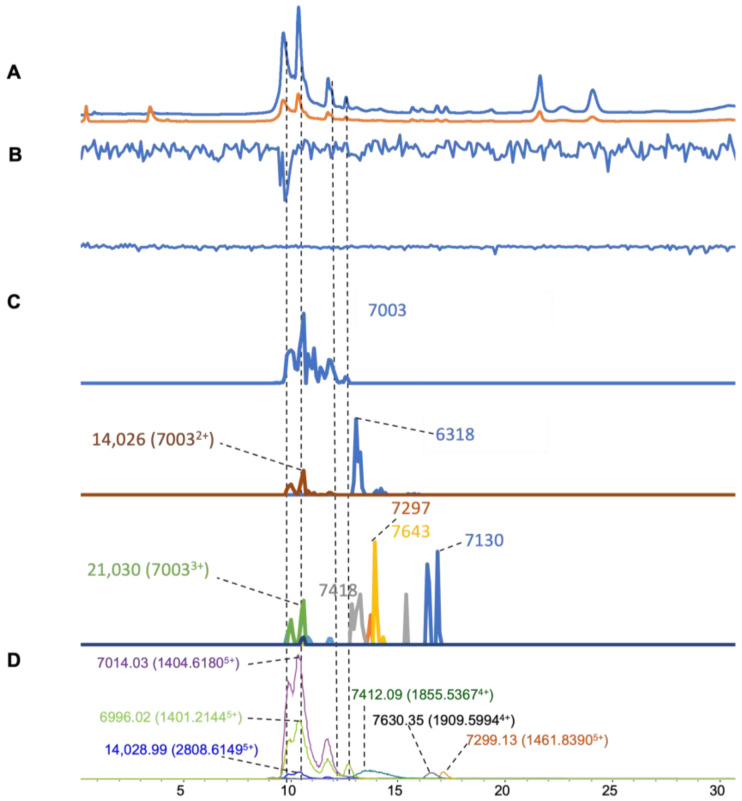
Integrated *O. hannah* venom results: Superimposed results of LC-UV, LC-(ESI)-MS, nanofractionated MALDI-MS data plotted chromatographically, and nanofractionated coagulation bioassay data plotted chromatographically. (**A**) LC-UV trace of separated snake venom at 220 nm (blue) and 254 nm (orange). (**B**) Coagulation bioactivity chromatograms representing anticoagulation (upper trace) and pro-coagulation activity (lower trace). (**C**) Chromatographically plotted MALDI-MS data of the identified toxins in the wells with nanofractionated toxins. For each toxin identified in different wells, the measured intensity from the MALDI data was plotted on the y-axis and the retention time of fractionation was plotted on the x-axis. As all the toxins were eluted over a series of subsequent wells, so-named MALDI-MS chromatograms of each toxin were obtained. Three sub-graphs are plotted in (C) with y-axis ranges of 0–6000 (high), 0–1000 (medium) and 0–80 (low). (**D**) Extracted Ion Currents (EICs) from the LC-ESI-MS data.

In the venom of *O. hannah* (Figure 7), six apparent masses with a high abundance were found from the ESI-MS data (Figure 7D), with a mass range of 7–14 kDa. For the MALDI-MS data, eight distinct masses with high intensity were deconvoluted (Figure 7C). Six masses were matched in both MS methods, which had a mass range between 6 and 14 kDa (Table 1). From the coagulation chromatogram data (Figure 7B), anticoagulation was observed around 10 min. It could be correlated to two ESI-MS masses of 7014.03 Da (7003 for MALDI-MS) and 14,028.99 Da (14,026 for MALDI-MS), which were anticipated to be a 3FTX and a PLA_2_, of which the PLA_2_ was tentatively assigned as the potential anticoagulation-causing toxin. A comparison of the molecular masses that we obtained to those of a proteomics study by Petras et al. [29], where the venom of *O. hannah* was studied using two venomics strategies (i.e., top-down and bottom-up), showed that a 3FTX (7001.05) was correlated to our mass data (7014.03 Da, 7003 for MALDI-MS). This toxin has also been reported in UniProt (ID, 3S11A_OPHHA).

### 2.7. Pseudonaja textillis

**Figure 8:**
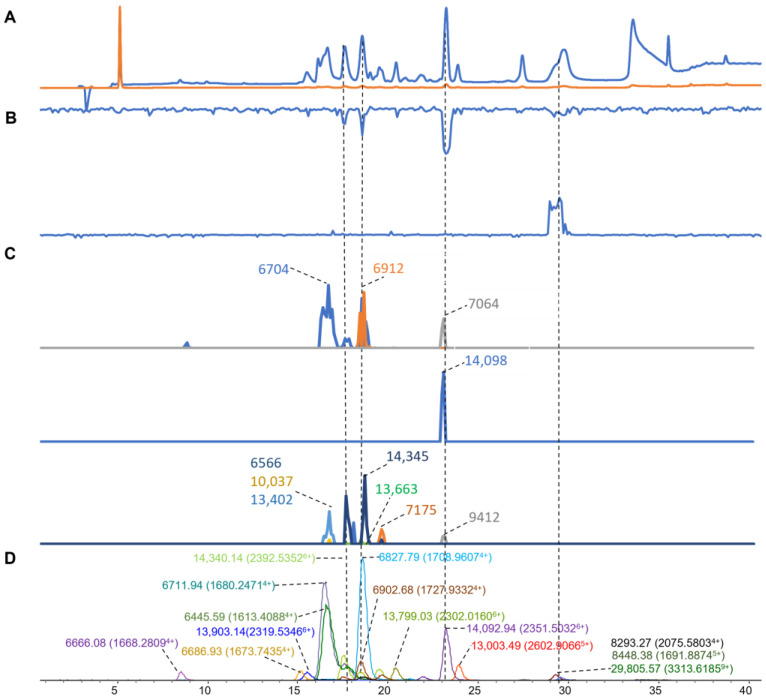
Integrated *P. textillis* venom results: Superimposed results of LC-UV, LC-(ESI)-MS, nanofractionated MALDI-MS data plotted chromatographically, and nanofractionated coagulation bioassay data plotted chromatographically. (**A**) LC-UV trace of separated snake venom at 220 nm (blue) and 254 nm (orange). (**B**) Coagulation bioactivity chromatograms representing anticoagulation (upper trace) and pro-coagulation activity (lower trace). (**C**) Chromatographically plotted MALDI-MS data of the identified toxins in the wells with nanofractionated toxins. For each toxin identified in different wells, the measured intensity from the MALDI data was plotted on the y-axis and the retention time of fractionation was plotted on the x-axis. As all the toxins were eluted over a series of subsequent wells, so-named MALDI-MS chromatograms of each toxin were obtained. Three sub-graphs are plotted in (**C**) with y-axis ranges of 0–3000 (high), 0–1000 (medium) and 0–80 (low). (**D**) Extracted Ion Currents (EICs) from the LC-ESI-MS data.

In the venom of *P. textillis* (Figure 8), sixteen distinct masses with a high abundance were found from the ESI-MS data (Figure 8D), for which masses in the mass range of 6–29 kDa were found. Eleven distinct masses with high intensity were deconvoluted from the MALDI-MS data (Figure 8C). Five of these masses were matched in both MS methods with a mass range between 6 and 14 kDa (Table 1). From the coagulation chromatogram data, we found both pro-coagulation and anticoagulation peaks resulting from nanofractionated toxins (Figure 8B). The venom of *P. textillis* is well-known for containing pro-coagulation activity [63,64]. For the results representing anticoagulation, three peaks were observed in the retention time frames of 18, 19, and 23 min, which corresponded to ESI-MS masses of 14,340.14 Da (for MALDI-MS 14,345), 6902.68 Da (for MALDI-MS 6912), and 14,092.94 Da (for MALDI-MS 14,098) Da, respectively. In contrast, Armugam et al. found a PLA_2_ toxin named Pt-PLA_2_ (Uniport ID: PA2A2_PSETE) that was isolated from *P. textilis* crude venom by a combination of gel filtration and RPLC [65] and was identified with an accurate mass of 14,348.32 Da and having apparent pro-coagulant activity. This toxin might match our accurate mass of 14,340.14 Da (for MALDI 14,345), but this toxin was observed as an anticoagulant based on our plasma coagulation results. Contrastingly, we observed pro-coagulation at 30 min of elution, which corresponded to two co-eluting small toxin masses (8293.27 and 8448.37) and one larger mass (29,805.57), deduced from the ESI-MS data.

### 2.8. Comparative Overview of the LC-ESI-MS, LC-MALDI-MS, and Coagulation Assay Results

The main objective of this study was to investigate the complementarity and overlap between LC-ESI-MS and LC-MALDI-MS results. The overlap between the ESI-MS and MALDI-MS data from our measurements was found to vary between 23.8 and 66.6% (calculated by the number of matched masses/the number of total masses found in ESI-MS or MALDI-MS, depending on which MS method retrieved more [medium to high intensity] masses x 100%). Most unmatched masses were found to have a relatively lower intensity. Regarding sample preparation for MALDI-MS after chromatographic separation, labor-intensive manual sample preparation is required, in addition to time-consuming manual data interpretation. In terms of data processing to produce the so-named MALDI-MS chromatograms, this manual process was also a time-consuming data processing endeavor, including the manual extraction of the *S*/*N* values for each toxin found in each well. The MALDI-MS method reported here thus appeared to not be the method of choice for rapidly acquiring and processing MS data for many venoms. Further, venom toxins can contain multiple isoforms with very similar molecular masses, which also poses a problem for MALDI-MS due to its mass error (for the instrumentation we used in our study, we used a mass error of <10 Da). In all the comparisons between ESI-MS and MALDI-MS, mass differences were observed for the overlapping results when taking the mass error of <10 Da into account. This mass error was, among others, based on a comparison study with MALDI-MS and ESI-MS by Escoubas et al. [66], which also gave this mass error for MALDI-MS of <10 Da. In addition, Newton et al. used a maximum *m*/*z* difference of 8 (<0.1%) for the mass fingerprinting of post-column fractions from the venom of the Indian red scorpion (*Mesobuthus tamulus*) obtained from ESI-MS and MALDI-MS data [54]. In a mass fingerprint analysis of *O. cayaporum* venom, they accepted less than 8 Da as an error when matching the results between ESI-MS and MALDI-MS [55]. In another MALDI-MS-based fingerprint identification study on enteric bacteria, a maximum error of 7 Da was used to compare the results from ESI-MS and MALDI-MS [56].

To be able to potentially visualize more bioactive toxins (such as toxins with low abundance and/or low bioactivity), higher injection concentrations can be considered, but column loadibility has to be taken into account in this regard. In our analytical methodology, mainly abundant toxins were found to be bioactive. This was also found in previous studies that used similar bioassays, such as that by Xie et al. [67,68]. When looking at other bioassays, such as online microfluidic acetylcholine binding protein assays for neurotoxicity profiling [69], low abundant highly potent toxins are also expected to be identified in neurotoxic venoms. To measure the potential bioactivity of lower abundant toxins, the injection of higher venom concentrations can be considered, in addition to two-dimensional LC where a semi-prep 1st dimension separation is used for low-resolution fractionation, followed by the analysis of these fractions one by one with the current methodology. Ligand fishing techniques as an initial bioactive toxin purification step can be considered, depending on the bioassay used [70]. For the venoms included in our study, venom proteomics studies have been performed and published. These proteomics studies comprehensively analyzed one or several venoms, focusing on the elucidation of the venom’s toxin composition. For instance, Chanda et al. analyzed venoms from *N. naja* and *N. kaouthia* from eastern India in 2018. They found 52 and 55 proteins for *N. naja* and *N. kaouthia*, respectively, of which 3FTxs and PLA_2_s were found in high abundance and in low abundance; SVMPs, nucleotidases, LAAOs, and CRISPs were also found [71]. Adamude et al. characterized *N. haje* venom by venomics methods, from which 14 protein families were identified, which consisted of 52% 3FTxs and 26% PLA_2_s, in addition to lower abundances of SVMPs (7%) and CRISPs (5%) [72]. *N. pallida* venom was analyzed by Kuna et al. using venom proteomics and found the venom to consist of 81% 3FTxs and 5% PLA_2_s, in addition to lower-abundance toxins (including VNGF (venom nerve growth factor), CRISPs, nucleases, LAAOs and SVMPs) [73]. For the venom of *N. atra*, venomics research revealed 84.3% 3FTxs and 12.2% PLA_2_s, as well as low abundances of CRISPs and SVMPs [74]. In *O. hannah* venom, 23 toxin families were identified by venom proteomics; 3FTxs and SVMPs were the most abundantly expressed toxins in the proteome, accounting for 43.0% and 24.4%, respectively, and followed by CRISPs (8.7%), LAAOs (5.7%), PLA_2_s (4.0%) and CVFs (2.8%) [75]. In a proteomics study focusing on *Pseudonaja textillis* venom, McCleary et al. identified 3FTxs, PLA_2_s, and Kunitz-type serine protease inhibitors as the main toxin families, followed by Snaclec, CRISPs, group C prothrombin activator subunits, Snaclec, SVMPs, and 5′-nucleotidases, by shotgun proteomics [76]. The proteomics studies discussed here are summarized in Table 2. The focus of this study was to develop an analytical methodology for chromatographic comparisons and alignments of MS datasets of Elapid venoms. This study did not include MS/MS experiments or proteomics research, and as such, only toxin masses and bioactivities were acquired. The inclusion of proteomics data in future studies would be a valuable progression of this research.

## 3. Conclusions

In the present study, we carried out chromatographic alignments to analyze nanofractionated venom toxins of several medically important elapid species (five *Naja* spp., *Ophiophagus hannah*, and *Pseudonaja textillis*) through two MS methods (i.e., ESI-MS and MALDI-MS) and included coagulation bioactivity screening data. Both MS methods presented complementary toxin identification capabilities for prominent toxins in relation to the coagulation bioactivity peaks observed. As for coagulation activity screening, most elapid venoms presented toxins with anticoagulation activity. For the toxins from *P. textillis* venom, pro-coagulation was additionally found. This study had some limitations, as follows: 1. This study only examined the major bioactivities and high-intensity toxins from the MS data. 2. The toxins analyzed were not quantified. Only the LC-UV data can be used to obtain very basic information on a toxin’s abundance based on the LC-UV peak area, which only can assess a toxin’s abundance in a venom at the level of low, medium, or high abundance. 3. The bioassay performance was useful for initial screening but did not provide potency or other quantitative parameters for the comparison of toxins. The most intense peaks do not necessarily correlate with the most active toxins in a venom. 4. Producing the chromatographic representation for the LC-MALDI-MS data is time-consuming and laborious. 5. In several cases, the insufficient separation of toxins resulted in (closely) co-eluting toxins, thereby making it difficult to pinpoint which exact toxin caused bioactivity. However, using the current methodology, we were able to narrow down the number of toxin masses correlated to the bioactivity peaks observed. 6. When using normal ESI-Q-TOF-MS, coupled with normal-bore LC separations, on average, sensitivity decreases when a toxin mass increases. 7. Toxins in snake venom can exist as multiple isoforms with very similar molecular masses, which poses a problem for the MALDI-MS instrumentation we used due to its mass error (for the instrumentation we used in our study, we allowed a mass error of <10 Da).

## 4. Materials and Methods

### 4.1. Reagents

Acetonitrile (ACN, UPLC/MS grade) and trifluoroacetic acid (TFA, MS grade) were purchased from Biosolve (Valkenswaard, the Netherlands). The water used in this study was obtained from a Milli-Q (MQ) plus system (Millipore, Amsterdam, the Netherlands). Calcium chloride (CaCl_2_) and phosphate-buffered saline (PBS) were purchased from Sigma-Aldrich (Zwijndrecht, the Netherlands). Bovine plasma (500 mL, Sodium Citrated, Sterile Filtered, Product Code: S0260) was purchased from Biowest (Nuaillé, France). α-cyano-4-hydroxycinnamic acid was obtained from Sigma-Aldrich (Zwijndrecht, the Netherlands).

### 4.2. Venoms

*Naja naja*, *Naja haje*, *Naja pallida*, *Naja atra*, *Naja kaouthia*, *Ophiophagus hannah*, and *Pseudonaja textillis* venoms were included in this study. Lyophilized *Naja* venoms, pooled from multiple animals, were provided by the Centre for Snakebite Research and Interventions Herpetarium (Liverpool School of Tropical Medicine, UK). *Ophiophagus hannah* venom, pooled from multiple animals, was provided by the National University of Singapore (NUS). *Pseudonaja textillis* venom, pooled from multiple animals, was provided by the University of Melbourne. Stock solutions of the crude venoms (1 ± 0.1 mg/mL) were prepared in water and then aliquoted and stored at −80 °C until use.

### 4.3. Nanofractionation Analytics

A Shimadzu SIL-30AC autosampler was used to inject venom solution (50 μL, 1 mg/mL) for separation on a 250 × 4.6 mm Waters Xbridge Peptide BEH300 C18 analytical column (3.5-μm particle size, 300-Å pore size) at 30 °C in a Shimadzu CTD-30A column oven. The eluate was sent to a Shimadzu SPD-M20A Prominence diode array detector (UV detector). The flow rate used was 0.5 mL/min and was controlled by two Shimadzu LC-30AD parallel pumps. Mobile phase A was composed of 98% H_2_O, 2% ACN, and 0.1% TFA, while mobile phase B was 98% ACN, 2% H_2_O, and 0.1% TFA. The gradient used was as follows: the concentration of mobile phase B increased to 13% in the first 5 min and then linearly increased to 50% within the time scope of 30 min, followed by a linear increase from 50% to 90% B within 3 min and then a 7 min isocratic elution at 90% B. Next, mobile phase B was decreased linearly to 0% in 1 min, and the column was then equilibrated for 5 min at 0% B. All the settings of the system were controlled by Shimadzu Lab Solutions software (v 5.117). After separation and UV detection, the eluate was split into a 1:9 ratio, of which the 90% fraction was transferred to a FractioMate^TM^ nanofractionator (VU, Amsterdam, the Netherlands) controlled by FractioMator software (v 1.0 VU, Amsterdam, the Netherlands). Fractions were collected at a resolution of 6 s/well onto transparent 384-well plates (F-bottom, rounded square well, polystyrene, without lid, clear, and non-sterile; Greiner Bio One, Alphen aan den Rijn, the Netherlands). The well plates with collected toxin fractions after separation were subsequently vacuum centrifuged to dryness overnight using a Christ Rotational Vacuum Concentrator (RVC 2–33CD plus, Zalm en Kipp, Breukelen, the Netherlands) operated with a −80 °C cooling trap. The dried plates were stored at −20 °C until MALDI-MS measurements (or bioassaying). In the figures, the y-axis ranges of the LC-UV data were from 0 to 700 for *N. kaouthia*, 0 to 400 for *N. naja*, 0 to 900 for *N. haje*, 0 to 600 for *N. atra*, 0 to 2750 for *N. pallida*, 0 to 900 for *O. hannah*, and 0 to 400 for *P textillis*, given in milli absorbance units (mAU).

### 4.4. Online (LC-)ESI-MS Measurements

After the flow split, a 10% split of the LC eluate was sent for MS detection (Impact II QTOF, Bruker Daltonics, Germany) with ESI in positive-ion mode at the mass range of 800 to 5000 *m*/*z*. The ESI source parameters were as follows: capillary voltage of 3.5 kV, source temperature of 200 °C, nebulizer at 0.8 Bar, and dry gas flow of 6.0 L/min. In-source collision-induced dissociation (CID) was set to 200 eV, and 1 average spectrum was stored per s. Bruker Compass software was used to control the instrument and analyze the data. For MS data processing, the total ion current (TIC) obtained from the recorded MS data was plotted. Extracted Ion Currents (EICs) obtained from the most abundant charge state of each toxin were plotted. The criteria for mass plotting were to plot *m*/*z* values from the intensity above 2000. For MS data processing, TIC was plotted based on the recorded MS data, with the y-axis ranging between 0 and 3 × 10^7^ signal intensity. The y-axis ranges of EICs were from 0 to 6 × 10^6^ for *N. kaouthia*, 0 to 9 × 10^6^ for *N. naja*, 0 to 1 × 10^7^ for *N. haje*, 0 to 3 × 10^6^ for *N. atra*, 0 to 2 × 10^7^ for *N. pallida*, 0 to 2 × 10^6^ for *O. hannah*, and 0 to 2 × 10^6^ for *P textillis*.

### 4.5. OffLine MALDI-MS Measurements

Well plates containing fractionated venom toxins were prepared for MALDI-MS by re-dissolving the contents of the wells in MQ water (10 μL). For MQ pipetting in the wells, a Multidrop 384 Reagent Dispenser (Thermo Fisher Scientific, Ermelo, the Netherlands) was used. After that, 1 μL of fractionated snake venom solution of the corresponding wells and 1 μL of the matrix (10 mg/mL α-cyano-4-hydroxycinnamic acid, dissolved in ACN:0.1%TFA in water (1:1)) were manually pipetted and aspirated three times on the MALDI plate (MTP 384 target ground steel BC, Bruker). Analysis was performed on an ultrafleXtreme MALDI-TOF/TOF mass spectrometer, and raw data were acquired using flexControl 3.4 software (both Bruker Daltonics, Bremen, Germany). The mass spectrometer was operated in linear positive-ion mode, with the following parameters: source 1, 20 kV; source 2, 18.9 kV; lens voltage, 6.5 kV; and laser repetition rate, 2 kHz. Prior to each analysis sequence of venom toxins, the system was first calibrated using a mixture of seven peptides in the range of 1000–3500 Da (Bruker Daltonics, Bremen, Germany). Spectra were recorded in the *m*/*z* range of 5000 to 55,000. Higher mass ranges (i.e., 50,000 to 150,000) were also measured, for which no venom toxins were detected. For each spectrum, 15,000 shots were accumulated from 30 different positions, with the laser power gradually increasing from 60% to 100%. For each well for which the corresponding LC-UV data suggested the presence of UV active compounds (i.e., venom toxins), the deconvoluted ions were analyzed and used to construct chromatogram-like graphs. This analysis was performed by plotting the *S*/*N* values of each toxin mass observed from each of the 6-s fraction’s recorded mass spectra against the corresponding retention time of fractionation. Since all toxins that eluted were fractionated over a series of adjacent wells, this way of plotting yields chromatographic representations for each toxin found with MALDI-MS, which can be compared to the EICs from the ESI-MS analyses. These graphs were not used in an absolutely quantitative manner but gave a visual representation of the high, medium, and/or low abundant signal ions of identified toxins. Specifically, for each spectrum and, thus, fraction, all relevant (i.e., significant) *m*/*z* values and corresponding signal-to-noise ratios (*S*/*N*) were processed using FlexAnalysis software (2.0 Bruker Daltonics, Bremen, Germany). MALDI-MS usually yields singly and doubly positively charged ions. It should be mentioned that with MALDI-MS, the charge state can sometimes not be deconvoluted from the spectra. This occurs when only one ion is found for a toxin. In these cases, it could not be determined if the ion is singly or doubly charged. However, when comparing the MALDI-MS data to the ESI-MS data, mass spectral information could be compared to elucidate a toxin’s charge state of the ion observed in MALDI-MS. When both the singly and the doubly charged ions were found in the mass spectrum, manual deconvolution was performed. The y-axis ranges of the MALDI chromatograms in the figures were 0–4000 (high) and 0–80 (low) for *N. kaouthia*; 0–6000 for *N. naja*; 0–10,000 (high), 0–600 (medium), and 0–100 (low) for *N. haje*; 0–8000 (high), 0–1000 (medium), and 0–20 (low) for *N. atra*; 0–10,000 (high), 0–600 (medium), and 0–60 (low) for *N. pallida*; 0–6000 (high), 0–1000 (medium), and 0–80 (low) for *O. hannah*; and 0–3000 (high), 0–1000 (medium), and 0–80 (low) for *P. textillis*.

### 4.6. Plasma Coagulation Assay

The bovine plasma stock was stored in 500 mL bottles at −80 °C. To avoid freeze–thaw cycles, upon first use, we aliquoted the bovine plasma in 15 mL CentriStar^TM^ tubes (Corning Science, Reynosa, Mexico), which we then stored at −80 °C. Each time bioassays were run, the required amount of bovine plasma was defrosted and centrifugated at 2000 rpm for 4 min prior to bioassaying. The bioassay protocol was performed based on previous research [23,46,47]. Briefly, a CaCl_2_ solution (20 mM, 20 μL per well) was pipetted onto a vacuum-centrifuge-dried 384-well plate with nanofractionated toxins using a Multidrop^TM^ 384 Reagent Dispenser (Thermo Fisher Scientific, Ermelo, the Netherlands). After 3 min, plasma at room temperature (20 μL per well) was also pipetted into the wells. The prepared plate was then immediately transferred to a Varioskan^TM^ Flash Multimode Reader (Thermo Fisher Scientific, Ermelo, the Netherlands) for kinetic absorbance measurements at 595 nm at 25 °C over 80 min. The resulting coagulation activity data measured in each well were processed into two coagulation scenarios, which were pro-coagulation (0–15 min slope readout) and anticoagulation (80 min endpoint readout). Pro-coagulation (y-axis from 0–2 absorbance units) and anticoagulation (y-axis from 0–0.8 absorbance units) responses were plotted on the y-axis, and the retention time of fractionation was plotted on the x-axis, resulting in coagulation chromatograms. These resulting two coagulation chromatograms were included in the figures that present the LC-ESI-MS and offline LC-MALDI-MS data. Positive peaks in the pro-coagulant chromatogram resulted from pro-coagulant toxins, whereas negative peaks in the anticoagulation chromatogram represented toxins with anticoagulant bioactivity.

### 4.7. Correlation of LC-ESI-MS (and LC-UV) Data with Offline LC-MALDI-MS and LC-Coagulation Bioassay Data

In our workflow, crude venom was separated by RPLC, with the resulting eluate divided into two sections via a flow split. After the flow split, 90% of the eluate underwent nanofractionation, while the remaining 10% was subjected to ESI-MS for analysis. The collected eluents were prepared for MALDI-MS measurements after evaporation by vacuum centrifugation. Alternatively, the dried plates could be used for post-column coagulation bioassaying. The LC-UV chromatograms were used to evaluate the chromatographic separations of the venoms, to easily inspect the elution of toxins at any given retention time, and as a starting point for the alignment procedure (which was needed as toxins go through the UV and to the ESI-MS detection and are fractionated, with slightly differing retention times). The retention time of each well in the 384-well plate could be calculated by the well number and collection time per well. As the procedure resulting in the well plates used for the plasma coagulation assay and MALDI-MS measurements was the same, there is no time difference for the bioassay chromatographic plotting and the MALDI-MS chromatographic plotting. The alignment procedure of the datasets was performed as follows: The retention time of a known negative bioactivity peak in the coagulation bioassay was compared to and aligned with the peak of this bioactive compound, also observed in LC-UV and ESI-MS by plotting the compound’s most abundant charge state ion in an EIC. The anticoagulant compound argatroban was used for the alignment procedure. By comparing retention times, the data sets were aligned, thereby making the retention time in each dataset the same. As the fractionation of argatroban on a well plate for bioassaying was performed in the same setup as the fractionation for MALDI-MS, the delay time of the bioassay chromatograms was the same as for the constructed MALDI-MS chromatograms. By matching EICs with the MALDI-MS data and with the bioactivity peaks in the bioactivity chromatograms through retention time and peak shape matching, the EICs that matched with plotted MALDI chromatograms were designated as the same toxin found for both MS techniques, and when they were matched with a bioactivity peak, they were designated as the probable accurate masses of the bioactive toxins. The y-axis values are not given in the figure; information on the y-axis dimensions is given above in Section 4 and in Table 1.

## Figures and Tables

**Table 1:** Comparison of the toxins found by LC-ESI-MS and by LC-MALDI-MS. In the table, the following columns are given: Venom species, RT (retention time), *m*/*z* (mass to charge) value of the most intense charge state, Charge of the most intense charge state, Accurate mass only found in LC-ESI-MS (Da), Accurate mass intensity only found in LC-ESI-MS, Accurate mass (Da) found in LC-MALDI-MS (bold), which matched with the mass found in LC-ESI-MS, Mass intensity of the MALDI-MS mass that was also found in the LC-ESI-MS data (i.e., the *S*/*N* signal extracted from Bruker software 2.0), Mass difference between LC-ESI-MS and LC-MALDI-MS, Mass only found in LC-MALDI-MS, Intensity (i.e., the *S*/*N* signal extracted from Bruker software) of mass only found in LC-MALDI-MS, and Percentage of the number of overlapped masses in both LC-ESI-MS and LC-MALDI-MS.

Venom Species	RT	*m*/*z*	Charge	Mass in ESI (Da)	Intensity	Mass in MALDI (Matched)	Intensity (*S*/*N*)	Mass Diff (MALDI-ESI) (Da)	Mass in MALDI (Unmatched)	Intensity (*S*/*N*)	Mass Matching Coverage (%)
*N. kaouthia*	7.8–10.3	1713.5188	4	6846.0296	118,960						38.4
	5.8–7.3	1721.0212	4	6875.0319	87,030	**6878**	2026	3	6250	53	
	10.3–12.3	1512.5336	5	7552.6119	28,926				12,500 (6250^2+^)	68	
	12.2–15.0	1955.8999	4	7815.5549	183,168	**7820**	1214	5	8348	3	
	16.7–18.3	1712.213	4	6840.8059	188,096						
	17.9–20.2	1523.7055	5	7608.4699	284,996						
	17.9–20.2	1921.1062	7	13,432.6	121,992	**13,434**	365	2			
	20.2–22.2	1678.085	4	6704.2948	678,428						
	20.2–22.2	1348.2753	5	6731.3139	169,332	**6721**	1860	10			
	22.3–23.6	1747.3826	4	6981.4859	37,924						
	23.6–26.0	1685.5989	4	6733.3464	552,306						
	23.6–26.0	1674.0763	4	6688.261	304,032	**6696**	3468	8			
	25.9–27.5	1715.3598	4	6853.3948	80,152						
*N. naja*	8.1–10.6	1720.0228	4	6872.0474	95,500						60
	8.1–10.6	1729.7932	4	6910.116	45,730	**6904**	445	6			
	8.1–10.6	1597.2398	4	6380.9144	49,120	**6380**	1770	0.9			
	10.4–14.1	1552.5054	5	7752.4698	22,620						
	10.4–14.1	1512.5363	5	7552.6249	25,002				13,936	48	
	14.1–19.8	1905.1154	7	13327.6723	10,810	**13,326**	4220	1.6	20,332 (10,165^2+^)	32	
	20.0–23.1	1685.0965	4	6732.3407	736,612	**6735**	1110	2	10,165	30	
	20.0–23.1	2266.7647	3	6795.2598	8040	**6790**	1540	5.2	13,591	66	
	20.0–23.1	1402.1052	5	7001.4724	79,996	**7007**	2460	5.6			
	23.1–24.9	1696.3524	4	6776.3613	492,842				13,558	22	
	23.1–24.9	1337.4707	5	6677.2952	16,360	**6675**	4352	2.2			
	24.8–26.5	1689.6003	4	6750.356	558,344	**6758**	625	8.3	13,517	36	
	26.4–32.4	1669.0753	4	6668.2556	930,222	**6678**	3124	9.8			
*N. haje*	14.3–19.2	1540.3371	5	7690.6252	259,700	**7694**	2060	4.6	5848	8	23.8
	14.3–19.2	1804.0359	4	7207.0973	45,036				6560	39	
	14.3–19.2	1809.2793	4	7227.0624	10,580	**7222**	1190	5	6393	529	
	19.1–21.8	1692.0586	4	6760.1881	1,300,398				9541	4	
	19.1–21.8	2707.0797	5	13,522.3358	42,858	**13,513**	311	8.7	9688	29	
	21.5–23.2	1713.0735	4	6844.2473	186,076				13,765	104	
	21.5–23.2	2336.032	3	7001.0381	776	**7006**	7298	5	20,290 (10,147^2+^)	81	
	23.1–27.8	1713.062	4	6844.2047	803,464				20,542 (10,260^2+^)	50	
	23.1–27.8	1367.2478	5	6826.1874	52,578	**6826**	6086	0.1	27,045 (7006^3+^)	26	
									29,076 (14,573^2+^)	8	
*N. atra*	7.1–10.0	1737.9997	4	6943.9542	296,046	**6947**	4086	3.1	5814	10	50
	9.8–13.3	1870.5805	4	7474.2789	6456	**7479**	232	4.8	6662	6085	
	14.8–15.7	1596.9376	4	6380.7067	22,144	**6381**	277	1.7	7005	236	
	14.8–15.7	1904.6295	4	7609.4763	7772	**7616**	1150	6.6	8387	15	
	13.4–14.8	1826.319	4	7297.2307	3260	**7305**	120	7.8	8852	12	
	19.2–21.3	2668.5028	5	13,328.4297	7130	**13,329**	869	0.6	10,172	18	
	21.1–27.3	2262.4411	3	6779.2931	46,420	**6771**	6367	–8.2	10,429	8	
	19.2–21.3	2696.2686	5	13,467.2678	1914	**13,474**	404	6.8	13,890	153	
*N. pallida*	12.4–15.0	1964.0992	4	7839.334	9400	**7832**	1132	−7.3	13,009	112	50
	12.4–15.0	1442.6444	5	7204.1603	261,398	**7206**	1869	1.9	13,470	450	
	18.4–20.2	1697.3461	4	6781.3384	644,342	**6792**	686	10.7	12,603	282	
	18.4–20.2	2665.1658	5	13,311.7823	19,900	**13,322**	1024	10.3	5637	49	
	19.6–23.1	1900.8443	7	13,283.7938	582,788				7468	4	
	19.6–23.1	1888.6947	7	13,204.7641	384,474	**13,212**	1049	7.3	5329	11	
	19.6–23.1	1747.5919	4	6975.3087	7046	**6973**	686	−2.3	7697	17	
	23.0–30.6	1705.5875	4	6814.3033	2,050,898				9244	25	
	23.0–30.6	1701.3316	4	6796.2811	33,470	**6793**	9138	−3.2	14,407	119	
	23.0–30.6	2728.5258	5	13,630.535	90,620	**13,632**	560	1.5	20,196	102	
	30.6–33.3	1680.0344	5	8389.4521	5064	**8404**	127	4.6			
	30.6–33.3	2099.7898	12	25,169.2375	12,436						
	30.6–33.3	1929.6506	11	21,206.4127	3546	**20,196**	102	10.4			
	33.3–36.3	1043.6935	7	7305.83	3444	**7313**	115	7.2			
*O. hannah*	9.2–11.4	1404.618	5	7014.0344	235,644	**7003**	4200	−11	21,030	28	66.6
	9.2–11.4	1401.2144	5	6996.017	101,660	**7003**	4200	4	7130	59	
	9.2–11.4	2808.6149	5	14,028.9908	12,904	**14,026**	270	−2.9	6318	834	
	12.4–17.9	1461.839	5	7299.1297	13,584	**7297**	19	−2.1			
	12.4–17.9	1855.5367	4	7412.091	15,948	**7418**	32	6			
	12.4–17.9	1909.5994	4	7630.3523	12,004	**7643**	65	12.7			
*P. textillis*	2.3–14.8	1668.2809	4	6666.0835	16,832				7064	1084	31.2
	14.6–16.1	2319.5346	6	13,903.1396	16,114				6566	19	
	14.6–16.1	1613.4088	4	6445.5897	19,406				10,037	5	
	16.0–17.4	1680.2471	4	6711.9367	149,532	**6704**	2319	−7.9	13,402 (6704^2+^)	29	
	16.0–17.4	1673.7435	4	6686.9284	194,924				7175	13	
	17.2–18.3	2392.5352	6	14,340.1415	49,090	**14,345**	61	4.9			
	17.2–18.3	1685.2454	4	6706.9143	25,428	**6704**	2319	−7.9			
	18.2–19.3	1708.9607	4	6827.7974	242,488				13,663	4	
	18.2–19.3	2391.5371	6	14,335.1533	38,600				9412	7	
	18.2–19.3	1727.9332	4	6902.6799	6986	**6912**	2049	9.4			
	20.0–22.7	2302.016	6	13,799.0286	24,990						
	22.6–23.8	2351.5032	6	14,092.9367	103,814	**14,098**	857	5.1			
	23.7–28.2	2602.9066	5	13,003.4925	31,024						
	23.7–28.2	3313.6185	9	29,805.5678	1994						
	28.2–32.7	1691.8874	5	8448.3763	11,332						
	28.2–32.7	2075.5803	4	8293.2742	6762						

**Table 2:** Overview of published proteomics studies dealing with the same venoms included in this study. Abbreviations: SVMPs: Snake venom metalloproteases, NGF: Nerve growth factor, LAAOs: L-amino acid oxidases, CRISPs: Cysteine-rich secretory proteins, CVF: Cobra venom factor, KPIs: Kunitz-type serine protease inhibitors, Snaclecs: Snake venom C-type lectin-like proteins, 5′-NT: 5′-nucleotidases, Vespryn: Venom PRY-SPRY domain-containing proteins.

Venom	Identified Toxin Families and Their Abundances	Number of Toxins Identified	Reference
*N. naja*	3FTxs (61.07%), PLA_2_s (20.17%), SVMPs (6.10%), NGF (3.13%), CRISPs (3.03%), and LAAOs (1.97%)	52	[71]
*N. kaouthia*	3FTxs (62.75%), PLA_2_s (17.48%), CRISPs (6.19%), SVMPs (4.53%), CVF (2.29%), and LAAOs (1.79%)	55	[71]
*N. haje*	3FTxs (52.14%), PLA_2_s (24.02%), SVMPs (7.2%), CRISPs (4.85%), LAAOs (3.63%), and CVF (2.93%)	57	[72]
*N. pallida*	3FTxs (81%), PLA_2_s (5%), CRISPs (3%), LAAOs (2%), NGF (2%), and SVMPs (1%)	N/A	[73]
*N. atra*	3FTxs (84.3%), PLA_2_s (12.2%), CRISPs (1.8%), SVMPs (1.6%), and NGF (0.1%)	N/A	[74]
*O. hannah*	3FTxs (43.0%), SVMPs (24.4%), CRISPs (8.7%), LAAOs (5.7%), Vespryn (5.7%), PLA_2_s (4.0%), and CVF (2.8%)	116	[75]
*P. textillis*	3FTxs (34.73%), PLA_2_s (I15.64%), KPIs (14.55%), prothrombinase complex (12.25%), Snaclecs (10.55%), SVMPs (5.7%), and CRISPs (5.4%), 5′-NTs (2.1%)	N/A	[76]

## Data Availability

Not applicable.
